# Radiative suppression of exciton–exciton annihilation in a two-dimensional semiconductor

**DOI:** 10.1038/s41377-023-01249-5

**Published:** 2023-08-24

**Authors:** Luca Sortino, Merve Gülmüs, Benjamin Tilmann, Leonardo de S. Menezes, Stefan A. Maier

**Affiliations:** 1 Chair in Hybrid Nanosystems, Nanoinstitute Munich, Faculty of Physics, Ludwig-Maximilians-Universität München, 80539 Munich, Germany; 2 Center for NanoScience, Faculty of Physics, Ludwig-Maximilians-Universität München, 80539 Munich, Germany; 3 Departamento de Física, Universidade Federal de Pernambuco, 50670-901 Recife-PE, Brazil; 4 School of Physics and Astronomy, Monash University, Clayton, VIC 3800 Australia; 5 The Blackett Laboratory, Department of Physics, Imperial College London, London, SW7 2BW UK

**Keywords:** Nanophotonics and plasmonics, Optical materials and structures, Sub-wavelength optics

## Abstract

Two-dimensional (2D) semiconductors possess strongly bound excitons, opening novel opportunities for engineering light–matter interaction at the nanoscale. However, their in-plane confinement leads to large non-radiative exciton–exciton annihilation (EEA) processes, setting a fundamental limit for their photonic applications. In this work, we demonstrate suppression of EEA via enhancement of light–matter interaction in hybrid 2D semiconductor–dielectric nanophotonic platforms, by coupling excitons in WS_2_ monolayers with optical Mie resonances in dielectric nanoantennas. The hybrid system reaches an intermediate light–matter coupling regime, with photoluminescence enhancement factors up to 10^2^. Probing the exciton ultrafast dynamics reveal suppressed EEA for coupled excitons, even under high exciton densities >10^12^ cm^−2^. We extract EEA coefficients in the order of 10^−3^, compared to 10^−2^ for uncoupled monolayers, as well as a Purcell factor of 4.5. Our results highlight engineering the photonic environment as a route to achieve higher quantum efficiencies, for low-power hybrid devices, and larger exciton densities, towards strongly correlated excitonic phases in 2D semiconductors.

## Abstract (web summary)

Enhancing light-matter interaction in 2D semiconductors coupled to Mie-resonant dielectric nanoantennas leads to the suppression of exciton-exciton annihilation, overcoming a fundamental limit for light emission in atomically thin materials.

## Graphical abstract

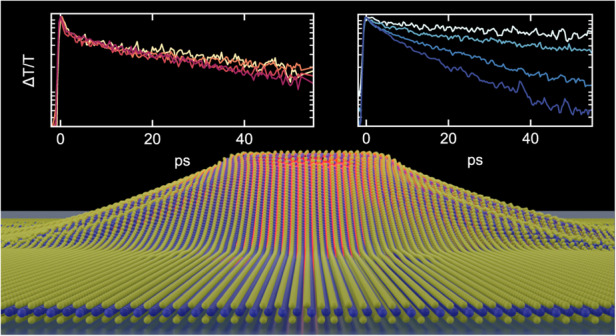

## Introduction

The Auger–Meitner effect in semiconductors is a scattering process where two charge carriers collide, resulting in a non-radiative decay via a mutual exchange of momentum^1^. It represents a major loss channel in optoelectronic devices, posing a fundamental limit on their quantum efficiency under high carrier densities. In particular, for low-dimensional semiconductors, quantum confinement restricts the momentum conservation rules of carriers and allows for stable Coulomb bound electron–hole pairs, or excitons. These can scatter via the mutual dipole-dipole interaction, in the form of exciton–exciton annihilation (EEA), yielding large scattering rates compared to bulk materials, as observed in quantum dots^2^, quantum wells^3^ and carbon nanotubes^4^. In the case of two-dimensional (2D) semiconductors, control and suppression of EEA is fundamental to unlock their potential for applications^5^. Transition metal dichalcogenides (TMDCs) emerged as the most promising family of atomically thin semiconductors for photonic applications^6^. Owing to large exciton binding energies above 200 meV, TMDCs optical properties are dominated by their excitonic response up to room temperature^7^, while at cryogenic temperatures TMDCs exhibit appealing properties, such as the presence of many body excitonic species^8^ and single photon emitters^9^. However, excitons in 2D TMDCs possess large Bohr radii, in the order of 1 nm, increasing their mutual interaction ranges and resulting in large EEA coefficients, reaching values larger than in any other semiconducting material^5^. EEA is thus observed even at relatively low exciton populations, setting a fundamental limit for the generation of high exciton densities in 2D semiconductors. Experimental techniques, such as time resolved luminescence or ultrafast transient absorption spectroscopy, have been employed to study EEA processes in atomically thin and bulk TMDCs^10–13^. EEA effect introduces a drastic change in the exciton dynamics, observed as a fast recombination process, in the order of a few picoseconds, which follows a quadratic dependence with the generated exciton population^11^. Recent works explored various approaches for suppressing EEA processes. For instance, by encapsulating TMDCs monolayers in hexagonal boron nitride^14^, extracting excess free carriers^15^, or the simultaneous application of strain and a gate voltage^16^. Notably, EEA processes in 2D semiconductors can also be harnessed to provide unexpected effects, for instance in generation of upconverted photoluminescence^17,18^, increased photocurrents^19^, and creation of negative mass excitons^20^.

An alternative approach to overcome the limitations imposed by EEA is offered by the integration of 2D semiconductors in nanophotonic architectures, tailoring the dielectric environment and the local density of states experienced by 2D confined excitons. The ability of 2D TMDCs to conform to underlying nanophotonic structures, and couple to the strong near field at their surfaces, have been demonstrated to enhance light–matter interaction in excitons^21–27^ and single photon emitters^28,29^, making them a promising material for hybrid nanophotonic devices. Resonant dielectric optical nanoantennas recently emerged as a novel platform to overcome the intrinsic losses of metal based plasmonic counterparts, while providing a new toolbox to tailor light–matter interaction at the nanoscale^30–34^. By sustaining the presence of both electric and magnetic types of optical resonances, multimodal interference of electromagnetic Mie modes in a single dielectric nanoantenna opens to higher degrees of control on light–matter interaction, from unidirectional scattering effects^35^ to suppression of far field emission^36^. This approach can be further extended to arrays of nanoantennas, or metasurfaces, for the manipulation of phase and amplitude of light in sub-wavelength dimensions and the physics of bound states in the continuum^37^.

In this work, we demonstrate the suppression of EEA processes via radiative rate enhancement, by coupling excitons in WS_2_ monolayers with Mie resonances of gallium phosphide (GaP) dielectric nanoantennas^38^. We show that the hybrid 2D semiconductor–dielectric nanoantenna system reaches an intermediate light–matter coupling regime and observe photoluminescence (PL) enhancement factors above 10^2^ compared to uncoupled monolayers, as well as a reduction of the PL lifetime, a signature of spontaneous emission rate enhancement. We then probe the exciton dynamics with ultrafast transient absorption spectroscopy. For uncoupled excitons, we observe the expected onset of non-radiative EEA as a fast recombination process in their dynamics^11^. On the contrary, excitons coupled to the near fields of GaP nanoantennas exhibit negligible changes in their dynamics over a broad range of excitation fluences. The combined effect of the enhanced absorption rate via near field coupling, and increased spontaneous emission rate via the Purcell effect, leads excitons to a higher probability of radiative recombination, rather than experiencing diffusion and non-radiative processes. This way, the overall EEA impact is reduced and at high exciton densities, and longer lived exciton populations can be sustained by the increased radiative rate. In the framework of a rate equation model, we extract the values of the EEA coefficient (*k*_A_) and found one order of magnitude lower values for WS_2_ excitons coupled to resonant nanoantennas, as compared to uncoupled excitons on glass substrate. Moreover, by comparing their ultrafast dynamics, we extract a Purcell factor (*F*_P_) of 4.5. This behavior goes against the phenomenological observation of decreasing EEA coefficients with longer exciton lifetimes^39^, highlighting enhanced light–matter interaction as a key for the suppression of EEA processes in 2D semiconductors. Our results demonstrate hybrid nanophotonics architectures as an attractive platform to engineer light–matter coupling with 2D materials and provide a route to overcome fundamental limitations induced by exciton–exciton scattering, enabling application of 2D semiconductors in photonic devices.

## Results

### Intermediate light–matter coupling regime in hybrid 2D semiconductor–dielectric nanoantennas

**Fig. 1:**
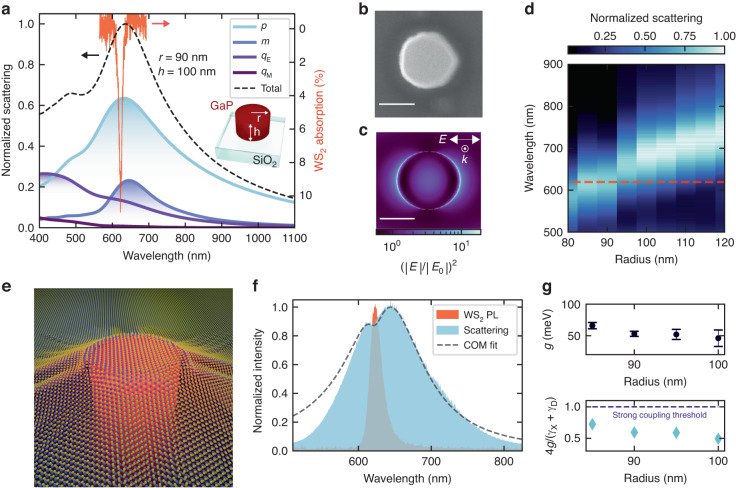
Hybrid 2D semiconductor–dielectric nanoantennas for enhanced light–matter coupling regime in 2D TMDCs. **a** Simulated scattering cross-section of a GaP nanoantenna with a radius of 90 nm and height of 100 nm (dashed black line), and absorption of the *1s*-exciton state of a WS_2_ monolayer on SiO_2_ substrate (red line). The scattering cross-section is analyzed with a multipolar expansion, where the respective weights of the electrical (*p*) and magnetic (*m*) dipoles, and the electrical (*q*_E_) and magnetic (*q*_M_) quadrupoles are shown. **b** Electron microscope image of a fabricated GaP nanoantenna. Scale bar 100 nm. **c** FDTD simulated electric field intensity, ${(| E| /| {E}_{0}| )}^{2}$, at the top surface of a GaP nanoantenna (*r* = 90 nm, *h* = 100 nm) at *λ* = 620 nm. Scale bar 100 nm. **d** Dark field scattering spectra of fabricated GaP nanoantennas for different radial dimensions. The dashed red line at 620 nm represents the resonance wavelength of WS_2_ monolayer excitons. **e** Illustration of the hybrid nanophotonic system composed of a WS_2_ monolayer coupled to a cylindrical GaP dielectric nanoantenna. **f** PL of nanoantenna coupled WS_2_ (in red) and dark field scattering (in light blue) of the GaP nanoantennas (radius 90 nm, height 100 nm). The scattering spectra is fitted with a coupled oscillator model (COM — dashed black line). **g** Upper panel: coupling strength (*g*) values extracted from the coupled oscillator model used to fit the scattering spectra of nanoantennas coupled with WS_2_ monolayers. Lower panel: ratio of the extracted coupling strengths values compared with the normalized strong coupling condition 4*g*/(*γ*_X_ + *γ*_D_) = 1

We select the geometry of the GaP nanoantenna to maximize the spectral overlap between the magnetic and electric dipolar Mie resonances and excitons in WS_2_ monolayers. Figure 1a shows the finite-difference time-domain (FDTD) numerical simulation of the scattering spectrum for a single GaP nanoantenna on a SiO_2_ glass substrate (dashed black line), with a radius of 90 nm and height of 100 nm. The red curve represents the experimental optical absorption of the *1s*-exciton state in a monolayer WS_2_ on SiO_2_ substrate, also referred to as A exciton in literature^7^. The scattering cross-section can be described with a multipolar expansion of the induced electromagnetic currents^37^, quantifying the individual contributions from the optical Mie resonances, respectively the electrical (*p*) and magnetic (*m*) dipoles, and the electrical (*q*_E_) and magnetic (*q*_M_) quadrupoles. We fabricated an array of optical nanoantennas by depositing thermally grown amorphous GaP on top of fused silica substrates, and patterned the thin film with conventional electron beam lithography and reactive ion etching techniques (see “Methods” for details). Figure 1b shows a top view electron microscope image of a fabricated cylindrical GaP nanoantenna on SiO_2_ substrate. At its surface, the nanoantenna confines and enhances the electromagnetic field intensity, ${(| E| /| {E}_{0}| )}^{2}$, defined as the ratio between the electric field amplitude of the scattered field by the antenna (*E*) and the normally incident field (*E*_0_). Figure 1c shows the numerical simulations for the enhanced near field region, resonant with the WS_2_ exciton energy at *λ* = 620 nm, recorded at the top surface of a single GaP nanoantenna. By tuning the radial dimension of the nanoantennas, we tailor the wavelength of the Mie resonances to match with the WS_2_ exciton wavelength. Figure 1d shows the dark field scattering spectra of the fabricated GaP nanoantennas array, in good agreement with the numerical simulations in Fig. 1a. As expected from Mie theory, increasing the resonator size shifts the Mie resonances to lower energies, crossing the WS_2_ exciton energy (dashed red line in Fig. 1d).

To probe the coupled system, we transfer the WS_2_ monolayer on top of the nanoantenna array with an all-dry transfer technique (see “Methods” and Supplementary Note I). Figure 1e displays an illustration of a monolayer WS_2_ transferred on top of a cylindric GaP dielectric nanoantenna on a glass substrate. The atomically thin layer stretches on top of the nanoantenna, in close proximity with the enhanced near field region, maintaining its quality and structural integrity as confirmed by Raman spectroscopy in our previous works^23,40^. Figure 1f shows the WS_2_ PL emission and the dark field scattering spectra of a hybrid nanoantenna covered with a WS_2_ monolayer. The scattering spectrum is modified by the presence of the atomically thin layer, in the form of a dip in correspondence to the PL exciton peak of the coupled WS_2_ monolayer (see also Supplementary Note II), indicating an enhanced absorption via the resonant coupling between excitons and Mie resonances^41^. We treat the nanoantenna’s optical resonances and WS_2_ excitons as damped coupled oscillators, and fit the scattering spectrum of the hybrid system with a coupled oscillator model (COM) in the form^41^:

1 $${\sigma }_{{\rm{scatt}}}(\omega )=A{\omega }^{4}{\left\vert \frac{({\omega }_{{\rm{X}}}^{2}-{\omega }^{2}-i\omega {\gamma }_{{\rm{X}}})}{({\omega }_{{\rm{D}}}^{2}-{\omega }^{2}-i\omega {\gamma }_{{\rm{D}}})({\omega }_{{\rm{X}}}^{2}-{\omega }^{2}-i\omega {\gamma }_{{\rm{X}}})-{\omega }_{{\rm{X}}}{\omega }_{{\rm{D}}}{g}^{2}}\right\vert }^{2}$$

where *γ*_X_ and *γ*_D_ are the exciton and antenna dipolar resonance linewidths, respectively, *ω*_X_ and *ω*_D_ the exciton and antenna resonance frequencies, *A* is a scaling constant, and *g* is the coupling strength constant. In Fig. 1g, we plot the extracted values of *g* (top panel) for WS_2_ coupled to different antennas with radius ranging from 85 to 100 nm. We found values in the range of 50–60 meV, comparable with similar hybrid architectures based on plasmonic nanoantennas^27^. We then compare the extracted values of *g* with the strong coupling condition satisfying that $2g \,> \frac{1}{2}({\gamma }_{{\rm{X}}}+{\gamma }_{{\rm{D}}})$^41^ (Fig. 1g, bottom panel). Due to the variation in linewidth of the fabricated antennas and the strain affecting the exciton resonance linewidth, we define a normalized value of the strong coupling condition as 4*g*/(*γ*_X_ + *γ*_D_) = 1, where the *γ*_X_ and *γ*_D_ values are extracted from the WS_2_ PL and nanoantenna scattering profile, respectively. For all the hybrid systems studied, we obtain values where *g* > 0.5, confirming the increased light–matter interaction of WS_2_ excitons coupled to optical Mie resonances, and placing our hybrid 2D semiconductor–dielectric system in the intermediate light–matter coupling regime.

### Radiative rate enhancement in coupled WS_2_ excitons

**Fig. 2:**
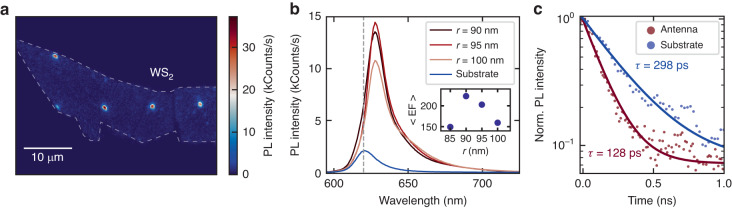
Photoluminescence enhancement and Purcell effect in WS_2_ monolayers coupled to GaP nanoantennas. **a** Map of WS_2_ monolayer PL on top of an array of resonant GaP nanoantennas. **b** PL signal of the WS_2_ on top of a GaP resonant nanoantennas with different radii (in red), and on top of the bare SiO_2_ substrate (in blue). The dashed line marks the unstrained exciton energy. Inset: experimental PL enhancement factor, $\left\langle {\rm{EF}}\right\rangle$, extracted from the integrated PL intensity. **c** Time-resolved PL traces from a WS_2_ monolayer coupled to a GaP nanoantenna with *r* = 90 nm (in red) and on the glass substrate (in blue), revealing a twofold reduction of the exciton decay dynamics ascribed to the Purcell effect. The data is fitted with a single exponential decay with offset (solid lines)

We further investigate the PL properties of coupled WS_2_ excitons by means of steady state and time resolved optical spectroscopy. Figure 2a shows the PL map of the monolayer transferred on top of a resonant GaP nanoantennas array. The sample is excited with a 530 nm, 140 fs pulsed laser, with a repetition rate of 80 MHz and average power of 14 nW. We scan the sample with piezoelectric stages, and record the PL intensity with an avalanche photodetector (see “Methods”). We observe more than one order of magnitude enhancement of PL emission when the monolayer is placed on top of the nanoantennas, owing to the interplay of enhanced light emission and absorption rates of the coupled nanophotonic system^23^. Figure 2b shows the spectra of WS_2_ on SiO_2_ substrate and on GaP nanoantennas with varying radius and fixed height of 100 nm. Here, the PL is sent to a monochromator and CCD camera, where a tenfold increase in the integrated PL intensity for coupled WS_2_ is observed. A maximum of PL is found for the nanoantenna with a radius of approximately 90 nm, as expected from the optimized spectral overlap between Mie modes and WS_2_ excitons (Fig. 1a). To fully capture the effect our hybrid nanophotonic platform we calculated the PL enhancement factor^42^, $\left\langle {\rm{EF}}\right\rangle$, resulting in values exceeding 200 (see inset in Fig. 2b and Supplementary Note III). Moreover, the PL peaks exhibit a redshift for coupled monolayers, consistent with the occurrence of tensile strain at the edges of the nanoantenna^40^. We found a maximum redshift of 21 meV, compared to the monolayers on flat substrate, corresponding to 0.4% tensile strain^43^, and exclude the presence of dark excitons or defect mediated emission, only observed in the cryogenic temperature PL emission of WS_2_ monolayers^7^.

We then studied the PL dynamics in coupled and uncoupled monolayer by collecting time resolved luminescence traces of with a streak camera setup (see “Methods”). As depicted in Fig. 2c, we observe a two-fold reduction of the decay lifetime for monolayers coupled to the nanoantenna near field, showing lifetimes of (128 ± 1) ps, compared to (298 ± 6) ps on bare substrate. Note, PL lifetimes in TMDCs are mainly limited by non-radiative processes, even at low fluences^7^, hindering the extraction of an effective value of the spontaneous rate enhancement. In our experiments, we employed a pump fluence of 120 μJ/cm^2^ to obtain appreciable signal to noise ratio, too large to neglect the impact of non-radiative processes in time resolved experiments^11^. To elucidate the role of strain in the PL experiments, we prepared a control sample, where a WS_2_ monolayer is transferred on top of SiO_2_ nanopillars with the same geometry and dimensions as the GaP nanoantennas. The nanopillar provides a deformation center, where strain is introduced in the monolayer, while lacking optical Mie resonances owing to its lower refractive index^29^. The experimental analysis of the control sample is presented in Supplementary Note IV. In strained WS_2_, we observe the presence of a strained exciton peak at the pillar sites, where a slight enhancement of the PL intensity could be detected. However, we observe no changes in the PL lifetime when compared to unstrained monolayers (Fig. S5b). This effect is consistent with previous reports where strain does not significantly impact the luminescence lifetime of WS_2_ monolayers^43^, we thus ascribe the reduction of PL lifetime in our hybrid 2D semiconductor–dielectric system to the Purcell effect.

### Ultrafast dynamics of coupled and uncoupled WS_2_ excitons

**Fig. 3:**
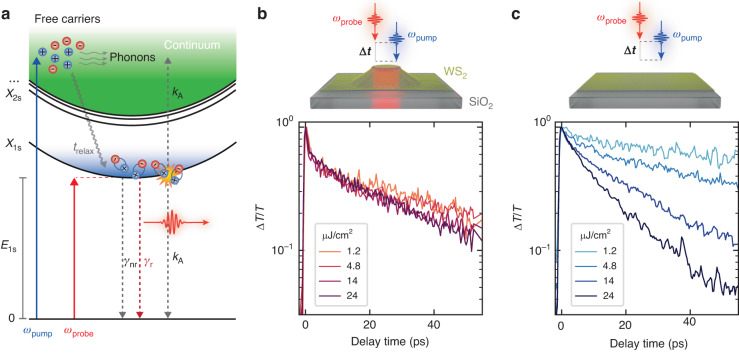
Ultrafast dynamics and exciton–exciton annihilation processes in coupled and uncoupled WS_2_ monolayers. **a** Schematics of the exciton formation and recombination dynamics under non-resonant excitation. A pulsed laser injects high energy electrons and holes which thermalize by losing their kinetic energy via exchange to phonons and scattering, relaxing to the *1s*-exciton state (*X*_1*s*_). The resulting exciton population recombines down to the ground state via radiative (*γ*_r_) and non-radiative (*γ*_nr_) recombination processes, or undergoes diffusion and scattering with other excitons resulting in exciton–exciton annihilation (*k*_A_). **b** Ultrafast differential transmittance (Δ*T*/*T*) of the WS_2_ exciton when coupled to the resonant GaP nanoantenna (radius 90 nm, height 100 nm), acquired for different pump beam fluences. **c** Fluence dependence of the differential transmittance of WS_2_ exciton on SiO_2_ substrate. The dynamics of uncoupled excitons exhibit the onset of EEA processes at fluences above 10 μJ/cm^2^, leading to a fast recombination of the exciton population compared to coupled excitons

To obtain additional insight on the exciton recombination dynamics in hybrid 2D semiconductor–dielectric nanoantennas, we investigated the coupled nanophotonic systems by means of non-degenerate ultrafast pump–probe spectroscopy (see “Methods” and Supplementary Note V). Figure 3a depicts a schematic of the excitons’ dynamics in a monolayer TMDC under non-resonant optical excitation. The absorption of high energy photons leads to the formation of free carriers in the high-lying bands, which undergo relaxation and formation of the exciton species via emission of phonons on a sub-100 fs timescale^44^. Due to the Wannier–Mott character, excitons diffuse in the crystal and the overall dynamics are described as the product of the spontaneous emission rate (*γ*_r_) and the non-radiative recombination rate (*γ*_nr_), e.g., from phonon, defects or carrier scattering, and EEA. The exciton population dynamics are described by the following equation:

2 $$\frac{{\rm{d}}N}{{\rm{d}}t}=G+D{\nabla }^{2}N-({\gamma }_{{\rm{r}}}+{\gamma }_{{\rm{nr}}})N-{k}_{{\rm{A}}}{N}^{2}$$

where *G* is the generation rate of excitons, *D* is the diffusion coefficient, and *k*_A_ is the EEA coefficient. When the exciton population density (*N*) is small, the recombination dynamics are dominated by the sum of radiative and non-radiative processes. As *N* increases, the quadratic term of the EEA starts to dominate the dynamics, leading to a fast decay of the photogenerated excitons^11^. Diffusion-related effects are to be expected to take place on a fast timescale, owing to exciton in-plane diffusion coefficients in the order of 200 nm^2^/ps^13^, and further increased by the effect of strain-induced exciton funneling^45^.

We compare the dynamics for excitons coupled with a resonant nanoantenna to that of a monolayer on bare SiO_2_ substrate. By resonantly probing the *1s*-exciton state, we investigate the impact of EEA processes in the exciton recombination dynamics as a function of the pump fluence, directly proportional to the photoexcited exciton population. The presence of strain in the hybrid system provides a favorable spectral separation of the coupled and uncoupled excitonic resonances (see Fig. 2b). Since the coupled WS_2_ area is smaller than the diffraction limited probe laser spotsize, we employed a narrow line width laser pulse and probe the transient absorption dynamics of coupled exciton resonances, with negligible signal from the uncoupled region.

Figure 3b shows the transient absorption dynamics for a WS_2_ monolayer deposited on a resonant GaP nanoantenna (radius 90 nm, height 100 nm), as a function of the pump beam fluence. We observe that the pump has negligible effects on the exciton lifetime, exhibiting minor changes for the range of fluences employed in our experiments, at the same time presenting a fast recombination process at zero-time delay, in the order of 1 ps, independent on the excitation fluence. Remarkably, we observe the absence of the onset of EEA processes, as expected for uncoupled excitons under fluences above 10 μJ/cm^2^^ 11^, further confirmed in WS_2_ deposited on the other resonant nanoantennas (see Supplementary Note VI). Note, that while the strain values extracted from the exciton redshift changes between nanoantennas, in the range 0.15–0.45%, the dynamics of coupled excitons are not significantly affected. These dynamics found for coupled excitons are in striking contrast with those observed in uncoupled monolayers. As shown in Fig. 3c, in the transient absorption signal of a monolayer WS_2_ on SiO_2_ substrate we observe clear changes in the exciton dynamics as a function of the pump fluence, in the form of the onset of a bimolecular recombination process, as expected from the role of EEA dominating the dynamics even under fluences as small as 10 μJ/cm^2^. As EEA processes can be neglected at the lowest fluence, the observed reduction of the lifetime in coupled excitons can be interpreted as the main effect of the Purcell effect, increasing the radiative recombination rate and reducing the overall exciton lifetime. In Supplementary Note VII, we compare the exciton dynamics at longer timescales, where the population of uncoupled excitons decays in ≈100 ps for excitation above 10 μJ/cm^2^.

We demonstrate the role of optical Mie resonances on WS_2_ excitons’ dynamics in Supplementary Note VIII, where we transferred WS_2_ monolayers on top of non-resonant GaP nanoantennas. As the Mie resonances are spectrally decoupled from the WS_2_ exciton (Fig. S10d), we observe negligible PL enhancement and the presence of an EEA onset in the ultrafast dynamics, as observed for monolayers on SiO_2_ substrates. These observations let us exclude possible effects due to doping from the GaP substrates, moreover, as the off-resonant antenna provides a larger contact area between GaP and WS_2_ compared to smaller resonant antennas, we conclude that the dielectric permittivity of the surrounding material does not significantly affect the EEA dynamics^46^. Changes in the surrounding dielectric material are expected to reduce the exciton binding energy^47^, which could be ascribed to the higher EEA coefficient observed on GaP compared to silica substrates (see Supplementary Note VIII).

### Radiative suppression of EEA via enhanced light–matter coupling

The ultrafast optical response of 2D semiconductors is described by the interplay of different effects determining their dynamics^48^. For instance, band gap renormalization and changes in the exciton binding energy, resulting in spectral shifts, or broadening of the exciton linewidth, via scattering and collisions, competing with Pauli blocking of photoexcited carriers. However, for excitation energies above the WS_2_ bandgap, as in our experiments, the ultrafast response is dominated by Pauli blocking^49^ and a fast sub-ps component traces the formation of excitons from the photogenerated free electron–hole pairs^50,51^. Large exciton binding energies hinder auto-ionization of resonantly excited excitons to high-lying states and, as shown in Supplementary Note IX, we further exclude thermal effects and defect-assisted recombination, following a linear dependence of the differential transmission signal modulation at zero time delay as a function of the pump fluence^52^. In our experiments, the dynamics are thus described within the picture of a thermalized population of the WS_2_ *1s*-exciton state.

**Fig. 4:**
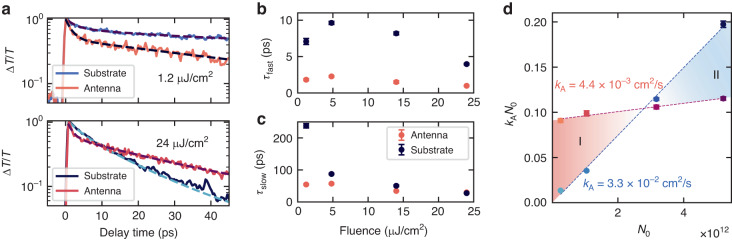
Suppression of exciton–exciton annihilation via radiative rate enhancement. **a** Differential transmittance traces for WS_2_ on substrate and on resonant GaP nanoantenna, excited with a pump fluence of 1.2 μJ/cm^2^ (top panel) and 24 μJ/cm^2^ (bottom panel). The data are fitted with a biexponential model (dashed lines). **b**, **c** Values of the fast, *τ*_fast_ (**b**), and slow, *τ*_slow_ (**c**), time component of the biexponential fit. The fast component is mostly unaffected by the pump fluence. For the slow component, at the lowest fluence we can exclude the effect of EEA processes and extract an enhancement of the spontaneous emission rate, or Purcell factor, equal to ${F}_{{\rm{P}}}={\tau }_{{\rm{slow}}}^{{\rm{off}}}/{\tau }_{{\rm{slow}}}^{{\rm{on}}}=4.5$. **d** Values of the EEA coefficient (*k*_A_) extracted from the differential transmittance values for WS_2_ coupled to a resonant nanoantenna (colored in red) and on SiO_2_ substrate (colored in blue), exhibiting a reduction of one order of magnitude for WS_2_ coupled to resonant nanoantennas

We fit the ultrafast exciton dynamics with a biexponential model (Fig. 4a) and extract the values of the fast and slow lifetime components, as shown in Fig. 4b. For all the coupled monolayers, we observe the presence of a fast sub-ps decay of the exciton population, independent of the pump fluence, and limited by the temporal resolution of our setup. As shown in Fig. S6, we compare the ultrafast dynamics of strained excitons to that of flat excitons on SiO_2_ substrate, where no sub-ps dynamics are observed in strained WS_2_. This fast component is observed predominantly in coupled excitons (see also Supplementary Note VI), and shows negligible dependence with the pump fluence. As previously reported, we ascribe it to the relaxation of free electron–hole pairs generated by high energy excitation, which rapidly forms the thermalized exciton population^53,54^. As this effect is enhanced only on resonant nanoantennas, changes in the fast time constants between coupled and uncoupled monolayer (Fig. 4b) could be related to an enhanced absorption rate via near field coupling with the optical Mie resonances^55^. At fluences of 1.2 and 4.8 μJ/cm^2^, we extract a constant ratio of the slow lifetime components on the antenna (${\tau }_{{\rm{fast}}}^{{\rm{on}}}$) and on the substrate (${\tau }_{{\rm{fast}}}^{{\rm{off}}}$) equal to ${\tau }_{{\rm{fast}}}^{{\rm{off}}}/{\tau }_{{\rm{fast}}}^{{\rm{on}}}=3.9$, while under higher fluences above 10 μJ/cm^2^, the ratio is reduced by the impact of the slower EEA processes. In contrast, for the slow lifetime component we observe a rapid reduction at higher fluences, as expected from EEA. Neglecting the effect of EEA and non-radiative processes at the lowest fluences, allows the extraction of a lower bound value of the enhancement of the spontaneous emission rate for coupled excitons. By comparing the slow lifetime components ratio at 1.2 μJ/cm^2^, we found an enhancement of ${F}_{{\rm{P}}}={\tau }_{{\rm{slow}}}^{{\rm{off}}}/{\tau }_{{\rm{slow}}}^{{\rm{on}}}=4.5$.

Finally, we obtain the EEA coefficient, *k*_A_, by fitting the transient absorption data for different pump fluences. We used a simplified rate equation model to extract the value of the number of normalized scattering events (*k*_A_*N*_0_), and compare the results with a linear model. In Supplementary Note X, we provide a discussion on the two models used, exhibiting negligible differences between the two fitting procedures. In Fig. 4d, we plot the extracted *k*_A_*N*_0_ values as a function of the injected exciton density (*N*_0_). From the slope of the linear fit, we extract the value of *k*_A_ = (4.4 ± 0.6) × 10^−3^ cm^2^/s in the case of coupled WS_2_ monolayer. Instead, for uncoupled monolayer on a glass substrate we found *k*_A_ = (3.3 ± 0.3) × 10^−2^ cm^2^/s, one order of magnitude higher than for the nanoantenna coupled WS_2_, and consistent with previous reports^12,51^. Moreover, we observe two regimes determined by the *k*_A_ values, denoted as Region I and II in Fig. 4d. In Region I, at lower exciton densities, coupled excitons exhibit a higher number of scattering events compared to the uncoupled case. This is a direct consequence of the fast time component in the dynamics, resulting in a larger drop of the overall modulation of the optical absorption, and thus a faster reduction in the first few picoseconds of the initial population *N*_0_ at the antenna position. However, above a certain density threshold, highlighted as Region II in Fig. 4d, *k*_A_*N*_0_ values for uncoupled excitons overtake the value for coupled ones, which, on the contrary, exhibit only a slight increase. Here, for uncoupled monolayers, the exciton recombination is dominated by EEA processes. As the coupled excitons have a larger radiative decay rate, more excitons are depleted radiatively and the overall effect of the EEA processes is reduced, directly impacting the instantaneous exciton density. This is also observed in the normalized differential transmission in Fig. 4a, where for high fluences the coupled systems sustains a higher excitonic population. The lower EEA coefficient in coupled excitons is a direct consequence of the quadratic dependence of *k*_A_ to the excitonic population shown in Eq. 2. We conclude that the EEA in 2D semiconductors can be suppressed by the faster radiative recombination, which reduces the probability for excitons to diffuse and participate in scattering processes, directly increasing the quantum efficiency and the possibility to realize longer lived high exciton densities in WS_2_ monolayers coupled to hybrid nanophotonic cavities.

## Discussion

In summary, we demonstrated the suppression of EEA processes in a hybrid 2D semiconductor–dielectric system by coupling excitons in monolayer WS_2_ with optical Mie resonances of GaP dielectric nanoantennas. The system reaches an intermediate light–matter coupling regime with PL enhancement factors above 10^2^, as a result of enhanced absorption and spontaneous emission rates. From the ultrafast exciton dynamics, we show the resilience of nanoantenna coupled excitons to sustain higher pump fluences without the onset of EEA processes. We extract one order of magnitude smaller EEA coefficients, together with a Purcell factor of 4.5. Owing to the increased radiative recombination rate, the exciton population is depleted faster via photon emission, suppressing the onset of EEA observed in uncoupled TMDCs monolayers. Engineering the photonic environment represents a novel opportunity to further reduce EEA processes in 2D semiconductors, for instance via integration with van der Waals metasurfaces^56^. Moreover, rationally designed hybrid nanophotonic systems based on 2D materials offer a vast toolbox for shaping and controlling light field at the nanoscale, for realizing higher spontaneous emission rates. Suppression of EEA via integration of 2D semiconductors with hybrid architectures can be extended to van der Waals heterostructures and Moiré systems, merging nanophotonics with many body physics and strongly correlated exciton phases^57^, and to novel hybrid platforms based on 2D and bulk van der Waals materials, as building blocks of optically resonant nanostructures^58,59^.

## Materials and methods

### Sample fabrication

GaP nanoantennas are fabricated with a top-down process using a combination of electron beam lithography (EBL) and reactive ion etching (RIE). First, 100 nm GaP and 80 nm SiO_2_ are grown on a glass substrate by sputtering. A double layer of polymethylmethacrylate resist and an additional conducting layer, to avoid charging effects, are spin coated on the sample. The resist is patterned with an EBL system, at 30 kV acceleration voltage, 20 μm aperture, dose of 330 μC/cm^2^, and then developed in a 1:3 solution of methylisobutylketone and isopropanol. 3 nm Ti and 30 nm Au thick layers are evaporated with an electron beam evaporator, acting as etching mask. We first removed the SiO_2_ layer via RIE and etch the gold with a standard gold etchant. The SiO_2_ left on the sample is used as a mask for the GaP RIE step, and is finally removed with an additional RIE etching, resulting in the desired GaP nanostructures on fused silica substrate. Monolayers of WS_2_ are exfoliated from a commercial single crystal (HQ Graphene) and deposited on top of the nanoantennas with an all-dry transfer technique based on polydimethylsiloxane^60^ in a home build transfer setup.

### Numerical simulations

FDTD simulations were carried out with a commercial software (Ansys Lumerical). The refractive index of the amorphous GaP film are extracted from ellipsometry measurements^61^.

### Optical spectroscopy

Dark field scattering experiments are performed in a commercial WiTec system with a broadband white light source. For PL and ultrafast spectroscopy experiments, we show a detailed schematic of the experimental setup in Supplementary Note V. The sample is mounted on a piezoelectric stage coupled to an inverted microscope system for mapping and fine tuning of its position. As excitation, we employ the frequency doubled output of a 180 fs pulsed tunable Ti:sapphire laser (Coherent Chameleon Ultra II) with a repetition rate of 80 MHz. The PL signal is collected with a monochromator and CCD detector (Princeton Instruments) or an avalanche photodetector (MDP) for PL mapping. The time resolved PL is acquired by directing the light to a monochromator and streak camera system (Hamamatsu). For pump–probe experiments, the frequency doubled Ti:sapphire laser is modulated at a frequency of 1990 Hz, with a mechanical chopper, and is used as the pump beam. The same laser drives a tunable optical parametric oscillator, which frequency doubled signal output is sent to an optical delay line and used as the probe. We excite the 2D semiconductor with laser pulses at 435 nm (2.85 eV), while resonantly probing the *1s*-exciton transition of WS_2_ monolayers at approximately 620 nm (2.00 eV), tuned to the maximum modulation of the WS_2_ response for each sample. The ultrafast dynamics are then recorded with a photodiode at the output of a grating monochromator, and with lock-in amplification. As the probe beam is resonantly exciting the exciton population, we carefully calibrated the impact of the probe energy by avoiding the presence of a fast decay peak in the response of the WS_2_. This is shown in Supplementary Note XI. We set the probe beam to a fluence of 1.5 μJ/cm^2^ in all our experiments. All the experiments were carried out at room temperature and in ambient conditions.

### Supplementary information

Supplementary Information

## Data Availability

The data that support the findings of this study are available from the corresponding author upon reasonable request.
