# Supplementary material for: Radiative suppression of exciton–exciton annihilation in a two-dimensional semiconductor
Source: Light Sci Appl. 2023 Aug 24;12:202. doi: 10.1038/s41377-023-01249-5 (PMC10449935; doi:10.1038/s41377-023-01249-5)
Supplement: Supplementary file 1 — Supplementary Information [file 41377_2023_1249_MOESM1_ESM.pdf]

# Supplementary Information for: Radiative suppression of exciton-exciton annihilation in a two-dimensional semiconductor

Luca Sortino,[1,2,*] Merve Gülmüs,[1] Benjamin Tilmann,[1,2]
Leonardo de S. Menezes,[1,2,3] and Stefan A. Maier[4,5,1,2]

[1] *Chair in Hybrid Nanosystems, Nanoinstitute Munich, Faculty of Physics, Ludwig-Maximilians-Universität München, 80539 Munich, Germany*

[2] *Center for NanoScience, Faculty of Physics, Ludwig-Maximilians-Universität München, 80539 Munich, Germany*

[3] *Departamento de Física, Universidade Federal de Pernambuco, 50670-901 Recife-PE, Brazil*

[4] *School of Physics and Astronomy, Monash University, Clayton, Victoria 3800, Australia*

[5] *The Blackett Laboratory, Department of Physics, Imperial College London, London, SW7 2BW, United Kingdom*

## SUPPLEMENTARY NOTE I: BRIGHT FIELD AND PL IMAGING OF THE HYBRID 2D SEMICONDUCTOR-DIELECTRIC NANOANTENNA SAMPLE

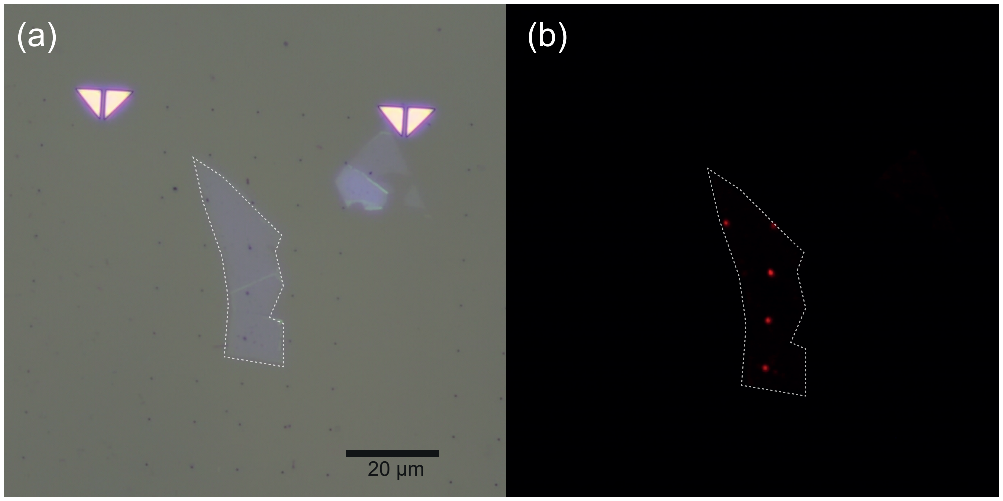


SUPPLEMENTARY FIGURE 1. (a) Optical microscope bright field imaging of a $WS_2$ monolayer transferred on top of an array of GaP nanoantennas. The dashed white line outlines the transferred monolayer. (b) PL imaging of the same sample, acquired in the same microscope with the use of spectral filtering [1], showing the enhanced $WS_2$ PL intensity at the nanoantenna positions.

## SUPPLEMENTARY NOTE II: DARK FIELD SCATTERING OF HYBRID 2D SEMICONDUCTOR-DIELECTRIC NANOANTENNAS

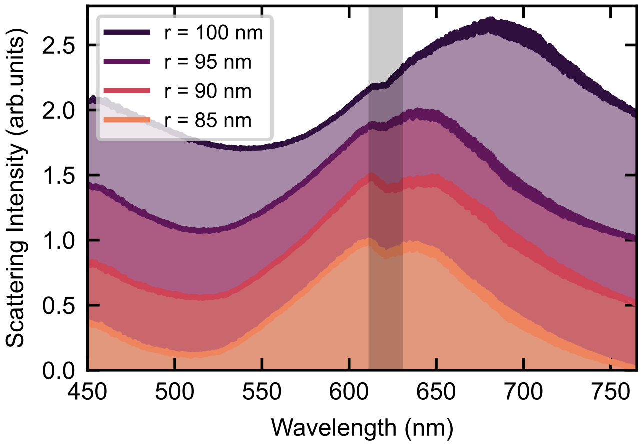


SUPPLEMENTARY FIGURE 2. Dark field scattering spectra of GaP nanoantennas with $WS_2$ monolayer deposited on top. The spectra are shifted vertically for display purposes. The gray shaded area corresponds to the $WS_2$ exciton resonance.

## SUPPLEMENTARY NOTE III: PHOTOLUMINESCENCE ENHANCEMENT FACTOR

The PL enhancement factor, $\langle EF \rangle$, is extracted from experiments as [2]:

$$\langle EF \rangle = \frac{I_{\text{on}}}{A_{\text{r}}} \left( \frac{I_{\text{off}}}{A_{\text{laser}}} \right)^{-1} \tag{1}$$

where, $I_{\text{on}}$ is the integrated PL intensity for $WS_2$ coupled to the nano-antennas, $I_{\text{off}}$ that of the uncoupled $WS_2$ on planar substrate. These are normalized on the PL excitation area, $A_{\text{laser}}$, defined by the excitation laser spot size, and the geometrical cross section of the cylindrical nanoantenna, $A_{\text{r}} = \pi r^2$.

We calculated the upper bound of the $\langle EF \rangle$ from finite-difference time-domain (FDTD) numerical simulations, following:

$$\langle EF \rangle \propto \frac{\sigma(\lambda_{\text{exc}})}{\sigma^0(\lambda_{\text{exc}})} \cdot \frac{F_{\text{P}}(\lambda_{\text{em}})}{F_{\text{P}}^0(\lambda_{\text{em}})} \cdot \frac{\eta(\lambda_{\text{em}})}{\eta^0(\lambda_{\text{em}})} \tag{2}$$

where $\sigma(\lambda_{\text{exc}}) \propto (|E|/|E_0|)^2$ is the near field intensity, $F_{\text{P}}(\lambda_{\text{em}})$ the Purcell factor, and $\eta(\lambda_{\text{em}})$ the directivity enhancement of the nanoantenna coupled dipole. These values are normalized over the ones obtained for the uncoupled case of a dipole on silica substrate. We placed the dipole 0.5 nm above the edge of the nanoantenna, in order to maximize the values of field enhancement and $F_{\text{P}}$ [2]. Figure S3a shows the near field values at the surface of the nanoantennas, taken at the top surface along the x-axis, as shown in the figure inset. Figure S3b shows the values of the Purcell factor along the same x-axis, for an in-plane dipole emitting at 620 nm along the x-axis, placed 0.5 nm above the nanoantenna's surface. For directivity simulations, we calculated the power emitted by the dipole source in the far field, integrated over the solid angle defined by the objective numerical aperture. We found values of $\eta/\eta_0 \approx 4$ for nanoantennas of radius of 100 nm, increasing with the antenna radius up to a value of $\eta/\eta_0 \approx 9$ for nanoantennas with radius of 85 nm. The product of these three ratios gives the upper bound of the $\langle EF \rangle$, shown in Figure S3c.

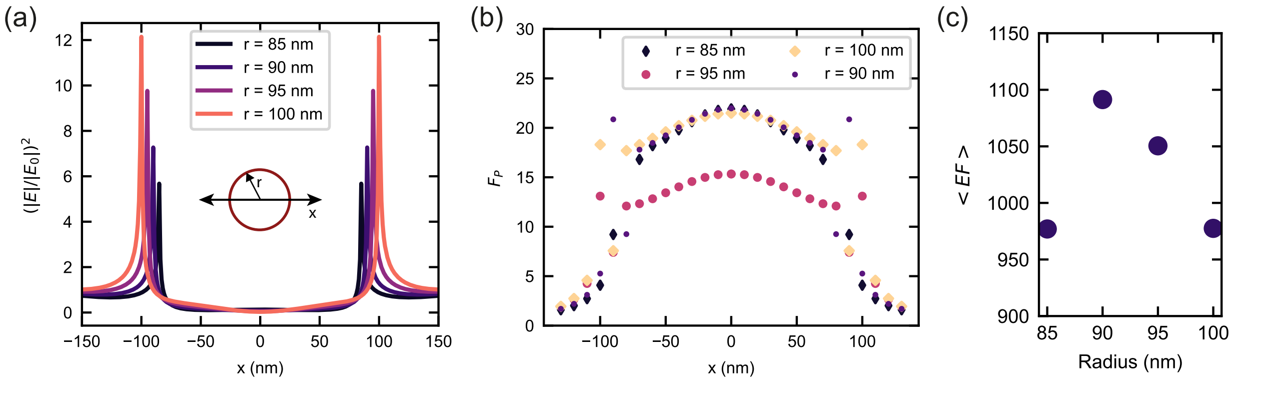


SUPPLEMENTARY FIGURE 3. (a) Profile of the numerically FDTD simulated near field intensity, taken along the x-axis as shown in the inset. (b) Purcell factor for a dipole at different position on the x-axis, placed 0.5 nm above the nanoantenna surface. (c) Maximum values of the $\langle EF \rangle$ calculated from numerical simulations.

## SUPPLEMENTARY NOTE IV: STRAINED $WS_2$ ON $SiO_2$ NANOPILLARS

We fabricated silica nanopillars to eluidate the role of strain in the PL and dynamics in strained $WS_2$ monolayers.

Figure S4a shows the schematic of the sample. The $SiO_2$ nanopillars are fabricated as follows. We employed a commercial Si wafer with a thermal $SiO_2$ layer of nominal thickness of 300 nm. We used a first etching step for thinning the oxide layer down to 100 nm, the target height of the nanopillars. We then use a combination of electron beam lithography and reactive ion etching (RIE) to pattern the silica nanopillars. Figure S4b shows an atomic force microscopy (AFM) profile of a fabricated nanopillar, revealing a height of 115 nm. The $WS_2$ monolayer is exfoliated and transferred on the nanopillars with an all-dry PDMS based transfer technique. Figure S4c shows the bright field image of the final sample. In Figure S4d is shown the PL map of $WS_2$ excited with a 180 femtosecond laser at 532 nm and average power of 6.5 nW. We observe a small increase of the integrated PL intensity when the monolayer is on top of the nanopillars.

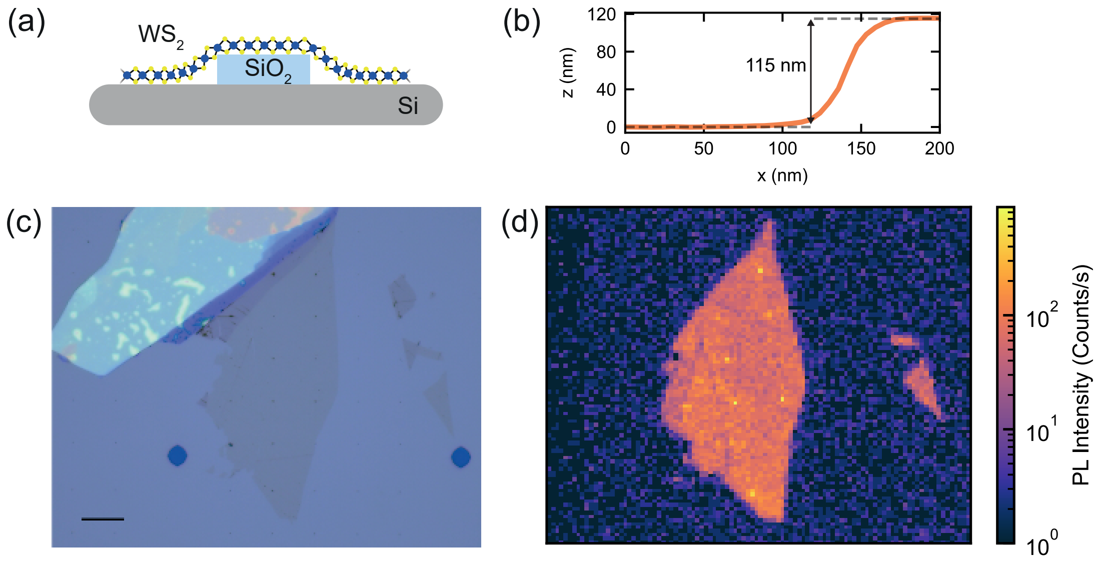


SUPPLEMENTARY FIGURE 4. (a) Schematics of the silica nanopillar sample with monolayer $WS_2$. (b) AFM scan of a single nanopillar's egde. (c) Bright field image of the $WS_2$ on silica nanopillar sample. Scale bar 10 $\mu$m. (d) Integrated PL intensity map of the silica nanopillar sample.

Figure S5a shows a representative PL spectrum collected on top of a single nanopillar, excited with a 532 nm laser pulse with average power of 1.4 $\mu$W. At low power densities, we observe the presence of a broad excitonic peak red shifted from the exciton energy of the unstrained $WS_2$ on the substrate (in gray), related to the effect of tensile strain on the exciton energy [3]. We probed the PL dynamics and observed negligible differences in the PL lifetimes (see Figure S5b), as previously observed in strained $WS_2$ [4]. From the fit of the PL spectra of different nanopillars, we extract the energy shift of the strained exciton resonance and calculate the relative tensile strain value. This is shown in Figure S5c, where we extract values between 0.4% and 1.1%. Note, these are larger values than the ones observed in our hybrid 2D semiconductor-GaP nanoantenna sample.

Figure S5d shows the PL emission as a function of the excitation power for the same nanopillar shown in Figure S5a. At low power densities, the strained exciton peak intensity is comparable or higher than the signal from the surrounding unstrained excitons. We ascribe this effect to funnelling, which pushes bright excitons towards the strained area [5]. Moreover, at higher power densities the unstrained exciton peak dominates the PL emission, together with the appearance of a broad peak at energies below 1.9 eV.

We further probed the ultrafast dynamics of flat and strained excitons. Figure S6 shows the differential reflection ($\Delta R/R$) for the $WS_2$ monolayer on top of a silica pillar with radius of 90 nm. We probed the exciton dynamics at two wavelengths, at 613 nm for the flat

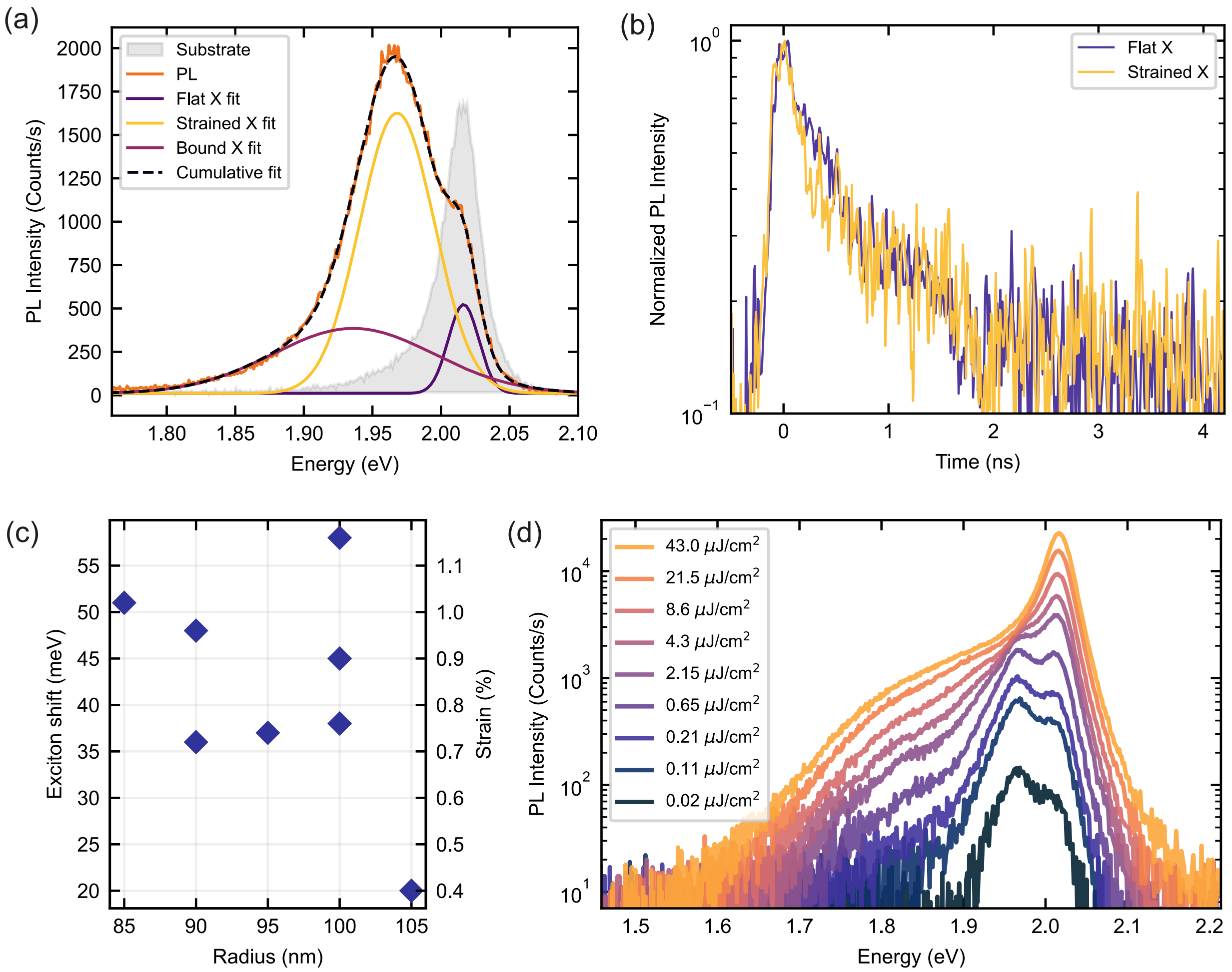


SUPPLEMENTARY FIGURE 5. (a) Representative PL spectrum of $WS_2$ monolayer transferred on top of a silica nanopillar. The spectrum is fitted with Gaussian peaks, exhibiting an unstrained flat exciton (X) peak at high energy, a broad red shifted peak ascribed to local strain, and a defect bound exciton peak. In gray, the PL emission of $WS_2$ on bare silicon substrate. (b) PL dynamics traces for the flat exciton and strained exciton shown in Figure S5a. The traces are obtained with femtosecond pulsed excitation at 532 nm and 5 $\mu$W average power. (c) Strained exciton red shift, and corresponding tensile strain value, extracted from the fit of the PL spectra of $WS_2$ on silica nanopillars with different radii. (d) Power dependence of the $WS_2$ PL emission shown in Figure S5a.

exciton and at 630 nm for the strained exciton (see PL in inset). The probe fluence was set at 1.2 $\mu$W cm$^{-2}$ and pump at 10.1 $\mu$W cm$^{-2}$. The high fluence conditions used to obtain a detectable signal in reflection measurements, are above the EEA onset. Indeed, for the flat exciton (blue curve) we observe a fast recombination dynamics related to EEA, while the strained exciton (red curve) we exhibit a slower recombination dynamics, with no fast component related to EEA. This exclude strain as the cause of the fast sub-ps dynamics observed when the $WS_2$ monolayers are coupled to GaP nanoantennas, as discussed in the main text.

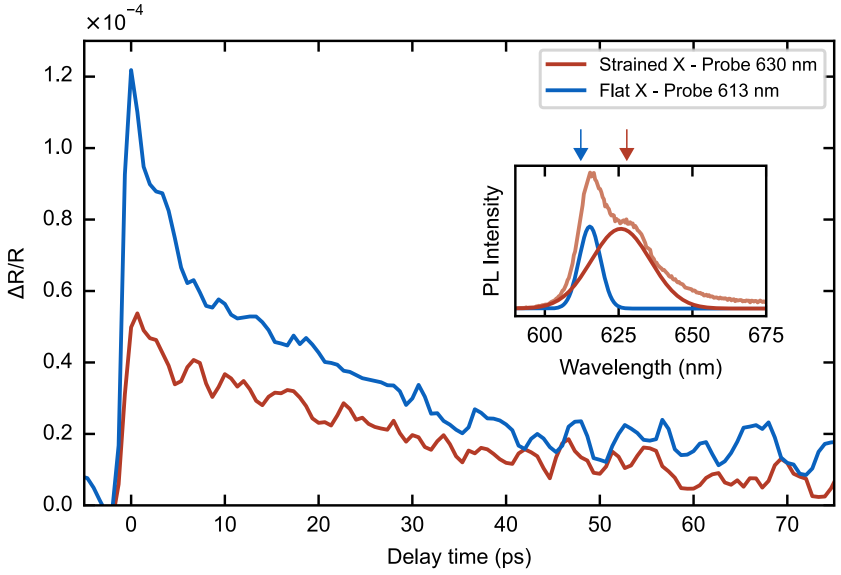


SUPPLEMENTARY FIGURE 6. Ultrafast transient reflection ($\Delta R/R$) for $WS_2$ excitons on silicon substrate (blue) and for strained excitons on silica nanopillar (red). Inset: PL spectrum of $WS_2$ on top of the silica nanopillar, the arrows indicate the probe wavelengths used in our experiments.

## SUPPLEMENTARY NOTE V: EXPERIMENTAL SETUP SCHEMATICS

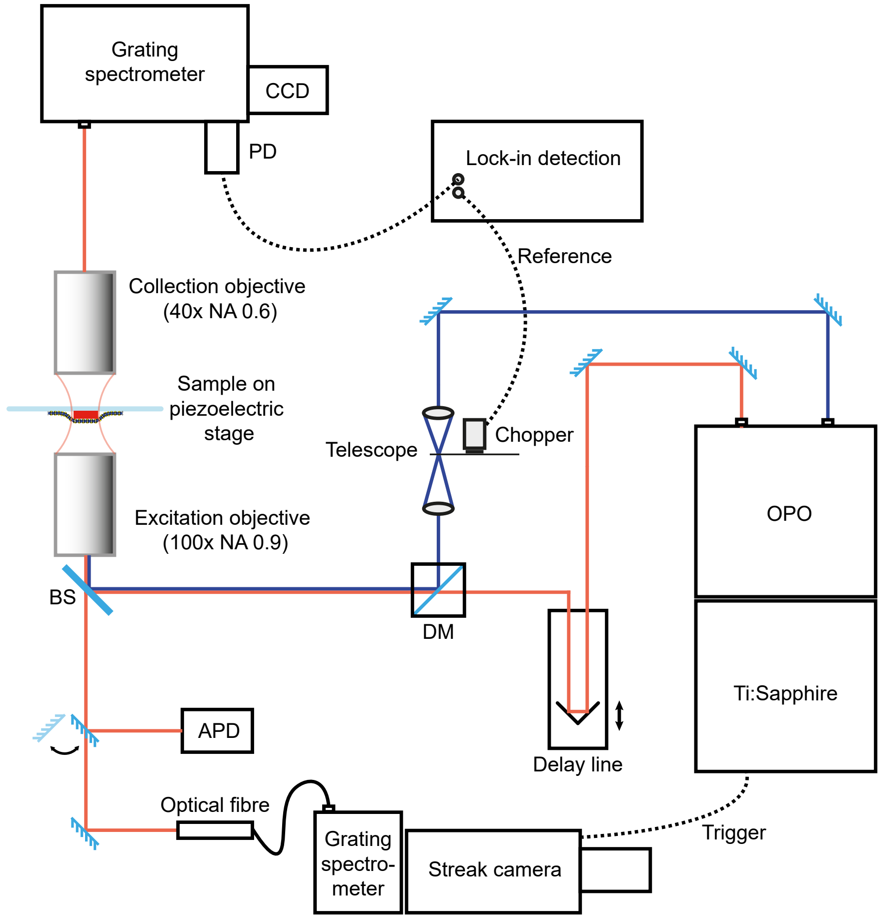


SUPPLEMENTARY FIGURE 7. Schematics of the experimental setup. The second harmonic signal (SH) generated from the fundamental wavelength of a Ti:Sapphire laser is used as the pump pulse. The same laser pumps an optical parametric oscillator (OPO), the tunable SH of the OPO is used as probe beam. A telescope system is used to expand the pump beam for maximizing the spatial overlap with the focused probe beam on the sample. Before reaching the sample, the pump is modulated with a mechanical chopper, placed at the focal point of the telescope system. The probe beam is directed to an optical delay line. The two beams are recombined with a dichroic mirror (DM) and sent to an optical inverted microscope, after a beam splitter (BS), where the sample is sitting on piezoelectric stages, and excited with high numerical aperture (NA) objective and low NA collection objective. Transmitted light from the sample is spectrally filtered and sent to a grating spectrometer and CCD camera for PL detection, or to a photodiode (PD) for pump-probe experiments with lock-in detection. The reflected light from the sample is sent to either an avalanche photodiode (APD) for PL mapping, or to an optical fibre coupled with a grating spectrometer and streak camera for time resolved PL measurements.

## SUPPLEMENTARY NOTE VI: ULTRAFAST DYNAMICS OF $WS_2$ EXCITONS COUPLED TO ADDITIONAL RESONANT GaP NANOANTENNAS

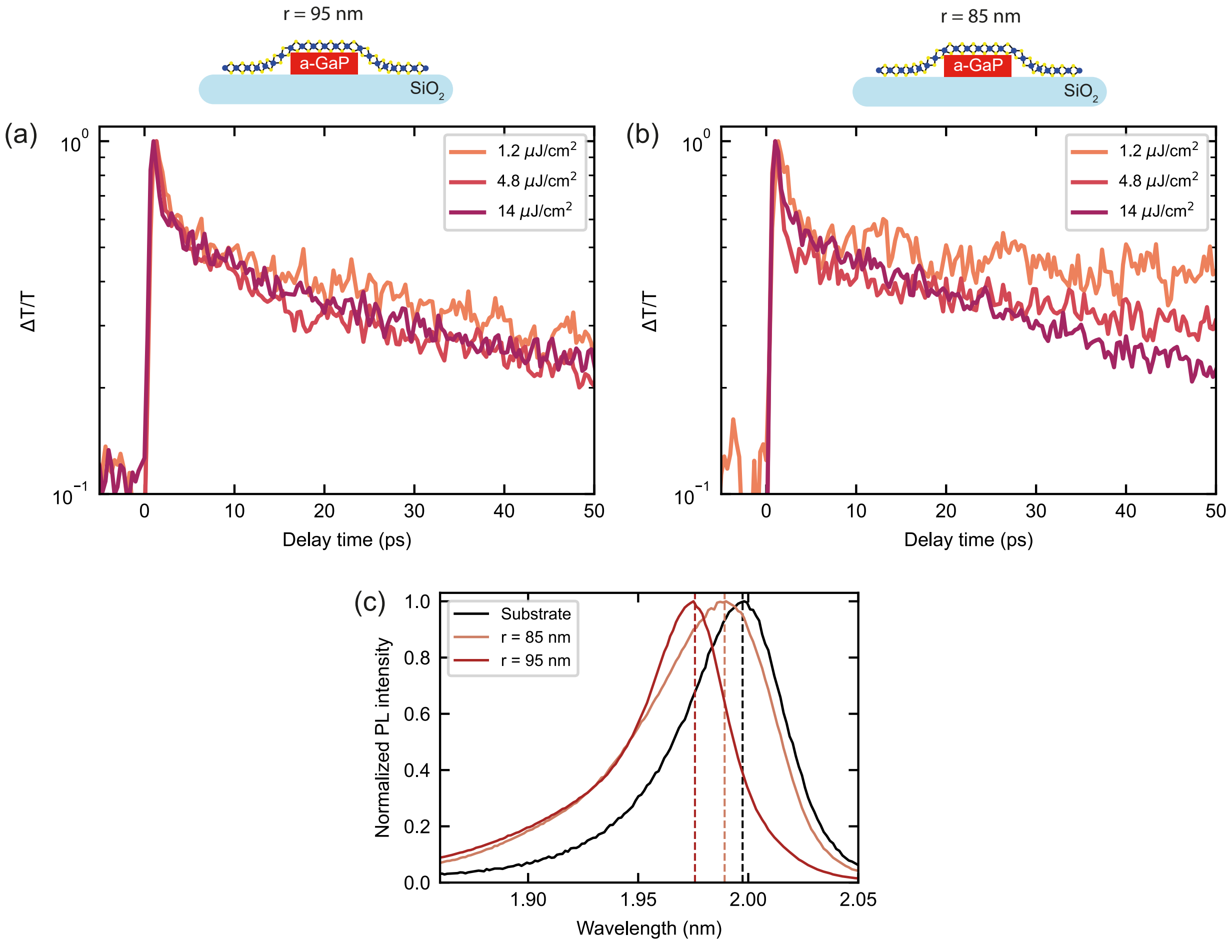


SUPPLEMENTARY FIGURE 8. (a) Power dependence of the differential transmittance ($\Delta T/T$) of the $WS_2$ excitonic state when coupled to a resonant antenna with radius of 95 nm. The extracted $k_A = (6.0 \pm 1.7) \times 10^{-3}$ cm$^2$ s$^{-1}$. (b) Power dependence of the differential transmittance ($\Delta T/T$) of the $WS_2$ excitonic state when coupled to a resonant antenna with radius of 85 nm. The extracted $k_A = (1.7 \pm 0.5) \times 10^{-2}$ cm$^2$ s$^{-1}$. (c) Normalized $WS_2$ PL spectra on flat substrate (black) and on antennas with radius of 95 and 85 nm. We estimate the strain introduced in the monolayer by fitting the PL data and comparing the exciton spectral shift to the value of unstrained monolayer on bare substrate. We extract a value of tensile strain of 0.47% for the $WS_2$ on the nanoantenna with radius of 95 nm, and of 0.16% when deposited on the antenna with radius of 85 nm.

## SUPPLEMENTARY NOTE VII: COUPLED AND UNCOUPLED EXCITON DYNAMICS AT LONG TIMESCALES

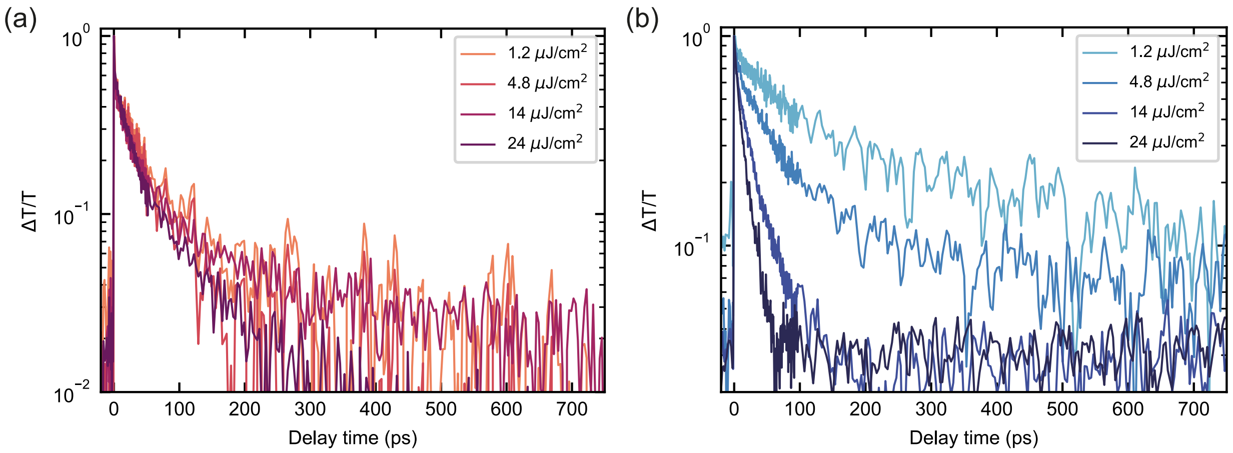


SUPPLEMENTARY FIGURE 9. Dynamics of monolayer $WS_2$ excitons when coupled to a GaP nanoantenna (a) and on bare silica substrate (b), showing timescale dynamics longer than those presented in Figure 3 in the main text.

## SUPPLEMENTARY NOTE VIII: $WS_2$ MONOLAYER COUPLED TO NON-RESONANT GaP NANOANTENNAS

We studied the effect of non-resonant GaP nanoantennas, with geometry of 50 nm height and radius of 320 nm, on the $WS_2$ PL and dynamics. Figure S10a shows the bright field image of a transferred $WS_2$ monolayer on arrays of non-resonant GaP nanoantennas. As shown in Figure S10b, we observe negligible PL enhancement when the monolayer is transferred on top of such nanoantenna, and a small redshift in the exciton peak corresponding to 0.05% strain. Figure S10c shows the PL dynamics, where a small increase of lifetimes at longer timescales is observed for monolayers on GaP. Figure S10d shows the dark field scattering of the studied sample, confirming the lack of spectral coupling between excitons and the optical resonances of the GaP nanoantenna, located at longer wavelengths above 700 nm. In Figure S10e we report the exciton dynamics probed with the same experimental conditions as described in the main text. The effect of EEA is clearly observed in the dependence with the pump beam fluence. From the ultrafast dynamics, we extract a rate of $k_A = (4.7 \pm 0.3) \times 10^{-2}$ cm$^{-2}$ s, consistent with the values observed for unstrained $WS_2$ on silica substrates [6].

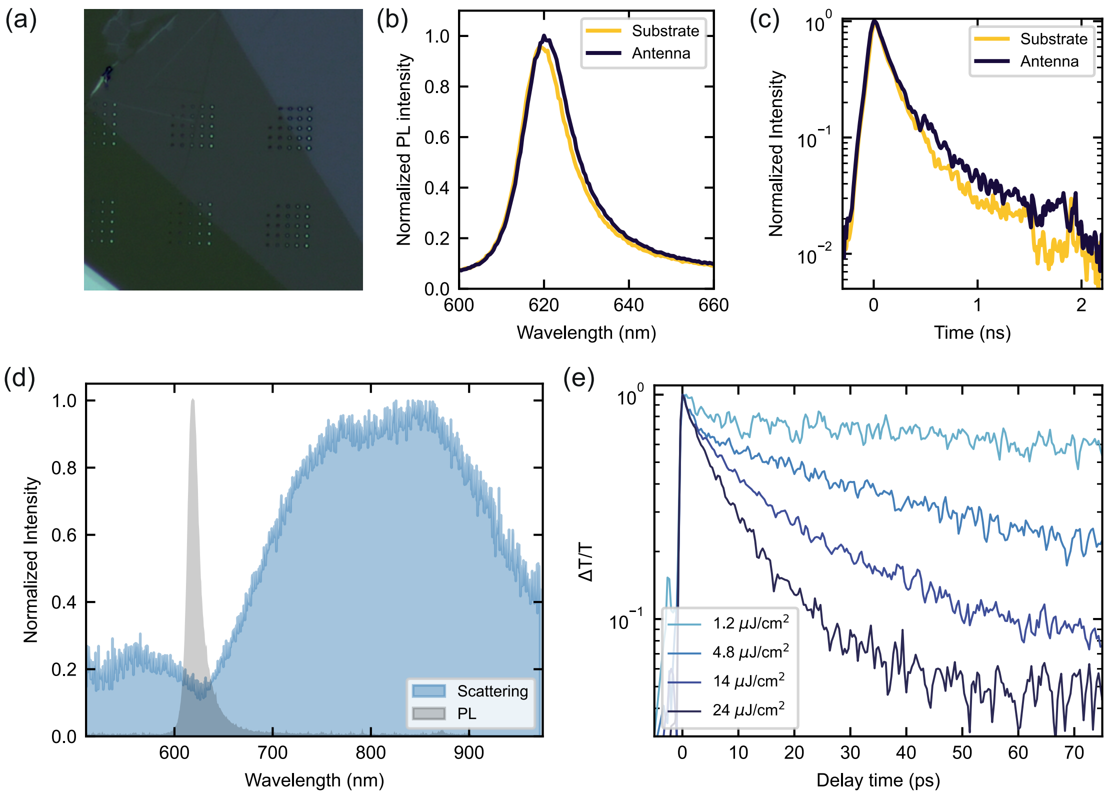


SUPPLEMENTARY FIGURE 10. (a) Bright field image of $WS_2$ transferred on top of GaP nanoantennas. (b-c) $WS_2$ PL spectra (b) and PL dynamics (c), on silica substrate (black) and on non-resonant GaP nanoantenna (h= 50 nm, r= 320 nm). (d) Dark field scattering of a GaP nanoantenna (h= 50 nm, r= 320 nm) and PL of the transferred monolayer $WS_2$ on top. (e) Ultrafast pump-probe exciton dynamics of $WS_2$ on non-resonant GaP nanoantennas.

## SUPPLEMENTARY NOTE IX: LINEAR DEPENDENCE OF $\Delta T/T$ AT ZERO TIME DELAY

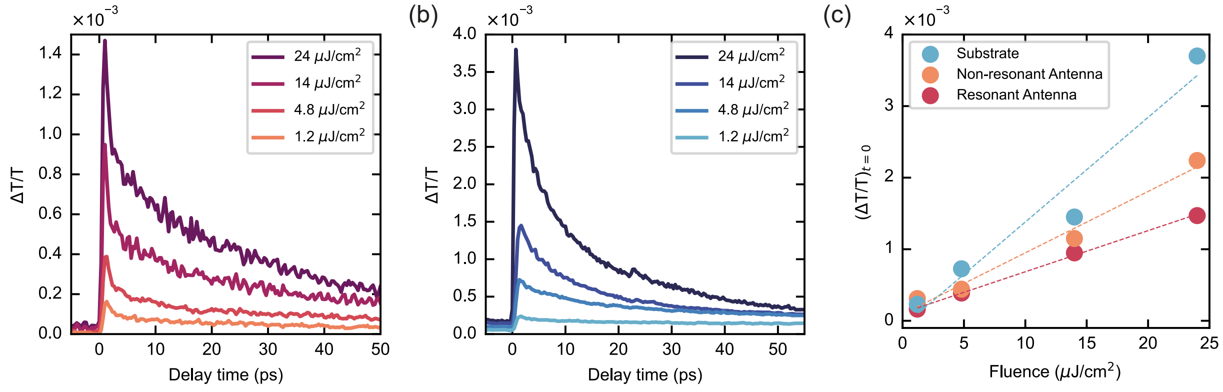


SUPPLEMENTARY FIGURE 11. (a) Transient absorption, $\Delta T/T$, of a $WS_2$ monolayer on resonant nanoantenna. (b) $\Delta T/T$ of a $WS_2$ monolayer on silica substrate. (c) Dependence of $\Delta T/T$ at zero time delay for $WS_2$ on resonant and on nonresonant nanoantennas, and on silica substrate. The lower modulation signal in the presence of the nanoantenna is related to the absorption and scattering of laser light by the nanostructure.

## SUPPLEMENTARY NOTE X: FITTING PROCEDURE TO EXTRACT EEA RATES

To extract the EEA coefficient ($k_A$) from the experimental data, we implemented two procedures [7, 8]. The first procedure is done by fitting the data with the following equation:

$$\frac{dN}{dt} = -k_A N^2 \tag{3}$$

This is a simplified case for Equation 2 in the main text. Figure S12a shows the fit to the ultrafast dynamics data for coupled and uncoupled excitons. Here, we neglect the diffusion term and, as EEA processes are significant only at the early times, long recombination dynamics do not impact significantly the extracted values of $k_A$ [8]. The values extracted for each fluence are then plotted with the generated exciton population, and the linear fit gives the EEA coefficient value. The second procedure consists in normalizing the zero-time delay modulation of the transient absorption, $(\Delta T/T)_{t=0}$, by the value of $N_0$, calculated for an absorption of 0.1% by $WS_2$ monolayer at the pump wavelength [9]. As shown in Figure

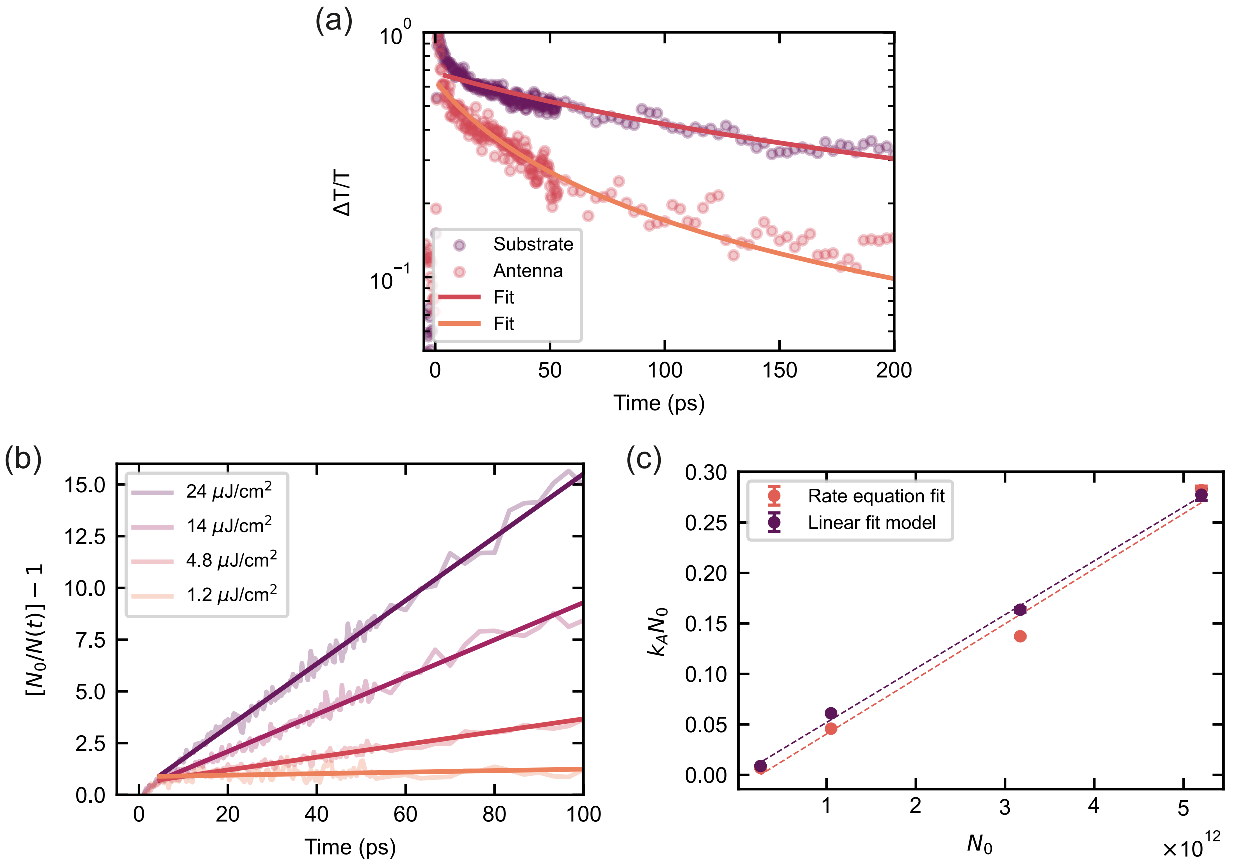


SUPPLEMENTARY FIGURE 12. (a) Differential transmittance for coupled and uncoupled excitons. The solid lines represent the relative fit using Equation 3.(b) Values of $[N_0/N(t)] - 1$ as a function of the time delay, and for different fluences, for a monolayer on silica substrate. From the slope of the linear fit we extract the $k_A N_0$ value. (b) Linear dependence of the $k_A N_0$ at different fluences, as a function of $N_0$. The slope of the linear fit gives the $k_A$ value.

S12b, the data are then fitted with the following equation:

$$\frac{N_0}{N(t)} - 1 = k_\mathrm{A} N_0 t \tag{4}$$

From the slope of the linear fit, we extract the values of $k_\mathrm{A} N_0$, at different excitation powers. As it is proportional to $N_0$, we plot the obtained values as shown in Figure S12b. From the slope of the linear fit we then extract the value of $k_\mathrm{A}$, which we found is not significantly affected by the chosen procedure.

## SUPPLEMENTARY NOTE XI: EFFECT OF RESONANT PROBE PULSE FLUENCE ON EXCITON DYNAMICS

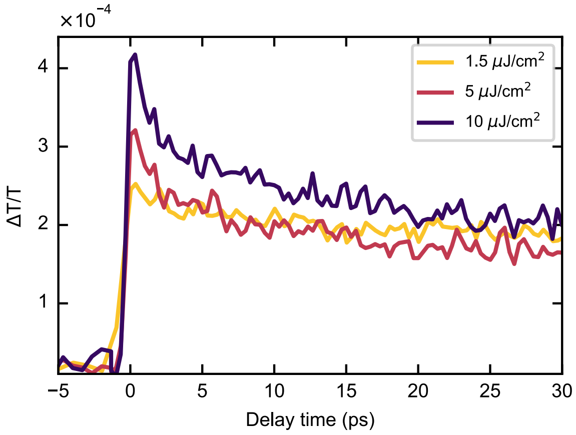


SUPPLEMENTARY FIGURE 13. Effect of the resonant 618 nm probe beam fluence on the exciton dynamics of $WS_2$ on bare substrate. Here, the fluence of the pump beam at 435 nm is kept constant at a value of 2.4 $\mu$J cm$^{-2}$. As the resonant probe promotes excitons in the excited state, we calibrated the beam power until the disappearance of the fast decay dynamics at zero-time delay, excluding the probe beam as a source of EEA in our experiments.

* luca.sortino@physik.uni-muenchen.de

[1] E. M. Alexeev, A. Catanzaro, O. V. Skrypka, P. K. Nayak, S. Ahn, S. Pak, J. Lee, J. I. Sohn, K. S. Novoselov, H. S. Shin, and A. I. Tartakovskii, Imaging of interlayer coupling in van der Waals heterostructures using a bright-field optical microscope, Nano Lett. **17**, 5342 (2017).

[2] L. Sortino, P. G. Zotev, S. Mignuzzi, J. Cambiasso, D. Schmidt, A. Genco, M. Aßmann, M. Bayer, S. A. Maier, R. Sapienza, and A. I. Tartakovskii, Enhanced light-matter interaction in an atomically thin semiconductor coupled with dielectric nano-antennas, Nat. Commun. **10**, 5119 (2019).

[3] L. Sortino, M. Brooks, P. G. Zotev, A. Genco, J. Cambiasso, S. Mignuzzi, S. A. Maier, G. Burkard, R. Sapienza, and A. I. Tartakovskii, Dielectric nanoantennas for strain engineering in atomically thin two-dimensional semiconductors, ACS Photonics **7**, 2413 (2020).

[4] I. Niehues, R. Schmidt, M. Drüppel, P. Marauhn, D. Christiansen, M. Selig, G. Berghäuser, D. Wigger, R. Schneider, L. Braasch, R. Koch, A. Castellanos-Gomez, T. Kuhn, A. Knorr, E. Malic, M. Rohlfing, S. Michaelis de Vasconcellos, and R. Bratschitsch, Strain control of exciton-phonon coupling in atomically thin semiconductors, Nano Lett. **18**, 1751 (2018).

[5] R. Rosati, R. Schmidt, S. Brem, R. Perea-Causín, I. Niehues, J. Kern, J. A. Preuß, R. Schneider, S. Michaelis de Vasconcellos, R. Bratschitsch, and E. Malic, Dark exciton anti-funneling in atomically thin semiconductors, Nat. Commun. **12**, 7221 (2021).

[6] L. Yuan and L. Huang, Exciton dynamics and annihilation in $WS_2$ 2D semiconductors, Nanoscale **7**, 7402 (2015).

[7] D. Sun, Y. Rao, G. A. Reider, G. Chen, Y. You, L. Brézin, A. R. Harutyunyan, and T. F. Heinz, Observation of rapid exciton–exciton annihilation in monolayer Molybdenum Disulfide, Nano Lett. **14**, 5625 (2014).

[8] N. Kumar, Q. Cui, F. Ceballos, D. He, Y. Wang, and H. Zhao, Exciton-exciton annihilation in $MoSe_2$ monolayers, Phys. Rev. B **89**, 125427 (2014).

[9] Y. Li, A. Chernikov, X. Zhang, A. Rigosi, H. M. Hill, A. M. van der Zande, D. A. Chenet, E.-M. Shih, J. Hone, and T. F. Heinz, Measurement of the optical dielectric function of monolayer transition-metal dichalcogenides: $MoS_2$, $MoSe_2$, $WS_2$ and $WSe_2$, Phys. Rev. B **90**, 205422 (2014).
